# Quality of Life Implications as a Consequence of Surgery: Limb Salvage, Primary and Secondary Amputation

**DOI:** 10.1080/13577140120099173

**Published:** 2001-12

**Authors:** Christine Eiser, Anne-Sophie E. Darlington, Chris B. Stride, Robert Grimer

**Affiliations:** 1 Department of Psychology University of Sheffield Sheffield S10 2TP UK; 2 Department of Psychology University of Durham Durham DH1 3LE UK; 3 Department of Work Psychology University of Sheffield Sheffield S10 2TP UK; 4 Royal Orthopaedic Hospital Woodlands Birmingham B31 2AP UK

## Abstract

*Purpose*. We investigated self-reported quality of life (QoL), body image and daily competence of patients as a consequence of limb salvage surgery (LSS), primary or secondary amputation, and the views of patients following secondary amputation.

*Patients*. Patients (n=37) had all been treated for osteosarcoma or Ewing's sarcoma in the lower limb.

*Methods*. QoL was measured by questionnaire. A separate interview to determine satisfaction with decision-making was conducted with those treated for secondary amputation.

*Results*. For the total group, QoL was below that expected from population norms. There were no differences in QoL between those undergoing LSS surgery compared with amputation. However, LSS reported better daily competence and were less likely to use a walking aid. For the total group, body image and daily competence were associated with better QoL.

*Discussion*. All these patients are at risk of compromised QoL following surgery. Our data are in line with previous work suggesting outcomes may be better for LSS compared with amputation. Following secondary amputation, most patients (80%) did not regret initial LSS treatment, but felt that the time gained allowed them to come to terms with subsequent surgery.


*Sarcoma* (2001) 5, 189–195

**ORIGINAL ARTICLE**

### Quality of life implications as a consequence of surgery: limb salvage, primary and secondary amputation

CHRISTINE EISER[1], ANNE-SOPHIE E. DARLINGTON[2], CHRIS B. STRIDE[3] & ROBERT GRIMER[4]

*[1] Department of Psychology, University of Sheffield, Sheffield S10 2TP, [2] Department of Psychology, University of Durham, Durham DH1 3LE, [3] Department of Work Psychology, University of Sheffield, Sheffield S10 2TP, [4] Royal Orthopaedic Hospital, Woodlands, Birmingham B31 2AP*

**Abstract**

*Purpose*. We investigated self-reported quality of life (QoL), body image and daily competence of patients as a consequence of limb salvage surgery (LSS), primary or secondary amputation, and the views of patients following secondary amputation.
*Patients*. Patients (n=37) had all been treated for osteosarcoma or Ewing's sarcoma in the lower limb.
*Methods*. QoL was measured by questionnaire. A separate interview to determine satisfaction with decision-making was conducted with those treated for secondary amputation.
*Results*. For the total group, QoL was below that expected from population norms. There were no differences in QoL between those undergoing LSS surgery compared with amputation. However, LSS reported better daily competence and were less likely to use a walking aid. For the total group, body image and daily competence were associated with better QoL.
*Discussion*. All these patients are at risk of compromised QoL following surgery. Our data are in line with previous work suggesting outcomes may be better for LSS compared with amputation. Following secondary amputation, most patients (80%) did not regret initial LSS treatment, but felt that the time gained allowed them to come to terms with subsequent surgery.

### Introduction

Survival rates for patients with bone cancer have increased substantially since the 1970s[1]. With modern treatment, survival rates in the order of 65% can now be achieved. Some 85% of patients are offered limb salvage surgery (LSS) and the remainder amputation. Primary amputation may be necessary depending on the exact location and the progressive state of the tumour[2], and is thought to offer good mobility but at the cost of compromised body image. For many patients, LSS is the treatment of choice, not least because it is possible to revert to amputation if necessary. Disadvantages of LSS include the increased risk of infections and breakages, and frequent hospitalisation for younger patients to lengthen the prosthesis. The advent of chemotherapy in the 1970s[3,4] has resulted in improvements in survival but can also involve lengthy and distressing treatments in the short-term, and compromised everyday competence, body image, work and social opportunities in the long-term. As a consequence it is important to consider the long-term psychological, functional and quality of life (QoL) outcomes.

There has been much argument about the definition and measurement of QoL[5], although the consensus points to a comprehensive concept which includes functioning across a range of domains, (e.g. physical, psychological and social). Attempts to distinguish between QoL for those treated by amputation or LSS have yielded inconsistent results[6]. Differences in physical functioning were reported by Rougraff et al.[7,8], with better outcomes for patients treated with LSS, but no differences in psychological outcomes have been found[7,8,9,10]. Patients treated for a bone tumour with LSS have reported poorer QoL compared with the normal population[11]. All these results need to be considered in relation to the considerable methodological problems involved in conducting QoL work with these patients[6].

Much of the published work does not address the very practical question involving patients' responses to an amputation after failed LSS (secondary amputation). One of the risks of LSS is an increased incidence of local recurrence and there is also the risk of infection or mechanical loosening, any of which can lead to failure of the prosthesis and thus the need for

Correspondence to: C. Eiser, Department of Psychology, University of Sheffield, Sheffield S10 2TP.

1357–714X print/1369–1643 online/01/040189–07 © 2001 Taylor & Francis Ltd
DOI: 10.1080/13577140120099173

secondary amputation. In these cases, previous experience with LSS may be valuable, enabling patients to come to terms with the disease and the possibility of future amputation. For others, failed LSS is an intense disappointment and simply represents wasted time. Clinically, it is vital to understand patients' views about failed LSS, in order to provide a quality service and to improve communication between surgeon and patient. If failed LSS with secondary amputation leads to a worse QoL than primary amputation, patients at high risk of failure might be better advised to undergo amputation in the first place.

There were four aims to the study. First, we attempted to extend our previous findings of compromised QoL in patients treated by LSS to include patients treated by either LSS or amputation. We compared QoL and general functioning in the total sample with population norms. Second, we considered the differences in QoL between patients undergoing amputation and those experiencing successful LSS. Third, we report the views of patients following secondary amputation, with particular emphasis on issues of decision making and subsequent adaptation to amputation. Finally, for the total sample, we made an attempt to determine which variables contribute most to QoL.

### Methods

Approval to conduct the study was obtained from the appropriate Ethics Committee. Patients were identified through medical records and invited to take part in a study concerning QoL following treatment for bone cancer. Patients were contacted by mail with information about the study and were asked to complete and return a reply slip indicating whether or not they wished to take part. Data were collected in patients' homes.

### *Patients*

All patients had been treated for osteosarcoma or Ewing's sarcoma in the lower limb at the Royal Orthopaedic Hospital in Birmingham between 1977 and 1995. We selected from our tumour database patients who had undergone amputation either primarily or secondarily and invited those who were free of disease and more than one year post-amputation to participate in this study. We also selected LSS patients who had been diagnosed and treated in the same year as the amputees with tumours of the same location. Thirty-seven patients (43% female) were recruited in this way. Three patients declined to take part in the study. There were no differences between treatment groups in sex, age or age at diagnosis as shown in Table 1.

### Measures

### *Demographics*

Patients completed a demographic questionnaire including information about education, employment, and marital status.

### *Quality of life*

The *SF36 Health Survey*[12] includes 36 items to assess QoL on eight different health related domains; physical functioning, physical role performance, emotional functioning, social functioning, pain, vitality, mental health, and general health. Higher scores indicate better functioning within the specific domain. Extensive work has been conducted to support the reliability and validity of this scale. In addition, norms for the British population are available[13].

### *Everyday competence*

Everyday competence[14,15] was measured by 32 items (e.g. putting on trousers; preparing meals). Patients were asked to rate each item on a five-point scale (impossible to do (1), extremely difficult (2), moderately difficult (3), a little bit difficult (4), not at all difficult (5)). Higher mean scores indicate less difficulty. Patients were also asked about their use of painkillers and walking aids.

**Table 1.** *Demographic data by patient groups*

| | Group 1 Primary amputation | Group 2 LSS | Group 3 Secondary amputation | Total sample |
|---|---|---|---|---|
| N | 12 | 14 | 11 | 37 |
| *Sex (no. females)* | 6 | 7 | 3 | 16 |
| *Age (years)* | | | | |
| Median | 32 | 30 | 32.5 | 31 |
| Range | 12–46 | 12–46 | 20–47 | 12–47 |
| *Age at diagnosis (years)* | | | | |
| Median | 19.5 | 16 | 19.5 | 19 |
| Range | 7–36 | 8–37 | 9–30 | 7–37 |
| *Time since diagnosis (years)* | | | | |
| Median | 7.5 | 7 | 12.5* | 10 |
| Range | 4–33 | 2–15 | 6–19 | 2–33 |

* $p < 0.05$

### *Body image*

The *Body Image Instrument*[16] was developed to assess body image in young survivors of cancer. It includes 28 items rated on a series of five-point scales (where 1 = strongly disagree and 5 = strongly agree) with higher scores indicating better satisfaction with body image.

### *Interview*

Patients treated with a secondary amputation were asked to take part in a semi-structured interview. The interview focused on the events leading up to the amputation, how the decision to undergo the amputation was made, and the patient's views about the way in which information was presented. Data was also collected on attitudes to undergoing an amputation after failed LSS.

### Treatment of data

Standardised questionnaires were scored as directed in the appropriate publications. Demographic data were summarised and described for each group. Student t-tests were used to determine differences on the domains of the SF-36 between the total group and population norms, and between the group of amputation patients and the group of LSS patients. For non-normally distributed data non-parametric comparisons were made between groups. T-tests and one-way analyses of variance (ANOVA) were used to compare general functioning between groups of patients. The interviews were transcribed and coded by two authors independently. Inter-rater reliability was established at 92%. A regression analysis was conducted to investigate predictors of QoL for the total group. Significance was accepted at the $p < 0.05$ level.

### Results

### *i) Comparison of QoL with population norms*

For the total group, the mean scores for the sample on the eight subscales of the SF-36 were compared with population norms[13] and are shown in Table 2. Significantly worse scores for patients compared with population norms were found for physical functioning, physical role performance, social functioning, vitality, pain and general health. However, the patients in the current sample did not report more emotional problems or poorer mental health relative to population norms.

A principal components factor analysis was performed based on the scores of the eight subscales of the SF-36. This yielded a single factor accounting for 62.2% of the variance. We therefore calculated a single index of QoL, which was used in the remaining analyses. A reliability analysis (indicating internal consistency) over the eight subscales yielded a Cronbach's alpha of 0.95. Data on the single QoL index were missing for six patients, because these patients did not complete all eight SF36 subscales.

### *Gender and age differences*

For the total group, women were more likely to use painkillers than men ($\chi^2 = 11.52$, $p < 0.01$). However, men reported better physical functioning (means = 59.0; 38.7; $t = -2.32$, $p < 0.05$). No other significant gender differences were found. The gender split in the three groups was not significantly different ($\chi^2 = 1.63$, $p > 0.05$), ruling out a sampling bias accounting for these differences. Everyday competence ($r = -0.36$, $p < 0.05$) decreased with chronological age.

### *ii) Amputation versus LSS*

Comparisons were first made between patients treated with an amputation (i.e. those with either a

**Table 2.** *Mean scores and Cronbach's alpha for outcome measures; comparison of total group scores with population norms, and comparison between subgroup scores*

| | Population norms[a] | Total group | Primary amputation | LSS | Secondary amputation |
|---|---|---|---|---|---|
| SF36 physical function | 92.0 | 50.0*** | 45.8 | 61.9 | 40.4 |
| SF36 physical role perf | 89.1 | 57.3*** | 69.9 | 57.7 | 47.5 |
| SF36 emotional function | 83.0 | 81.2 | 81.8 | 75.7 | 86.7 |
| SF36 social function | 83.9 | 62.6*** | 56.0 | 67.6 | 64.4 |
| SF36 vitality | 62.5 | 50.2* | 51.7 | 56.9 | 39.5 |
| SF36 pain | 80.8 | 62.7** | 62.0 | 67.6 | 57.8 |
| SF36 mental health | 74.7 | 69.9 | 71.6 | 74.8 | 62.2 |
| SF36 general health | 76.7 | 62.4** | 58.0 | 65.2 | 64.2 |
| QoL Index[b] | | | 64.4 | 66.8 | 57.6 |
| Social anxiety | | | 7.4 | 5.1 | 7.7 |
| Everyday competence | | | 3.8 | 4.1 | 3.5 |
| Body image | | | 84.0 | 90.4 | 80.8 |

[a] Population norms from Jenkinson, Coulter & Wright (1993)
[b] Missing data for six patients
* $p < 0.05$, ** $p < 0.01$, $p < 0.001$

primary or secondary amputation; n = 23), and those successfully treated with LSS (n = 14). There were no differences between the two groups (amputation mean = 61.2, LSS mean = 66.7) on the QoL index ($t = -0.62$, $p > 0.05$), nor in terms of level of education, type of employment or reported use of painkillers. A comparison of demographic variables *age*, *age at diagnosis* and *time since diagnosis* did not show any significant differences between the two groups.

Patients treated with LSS reported better everyday competence (mean = 4.2) than patients with an amputation (mean = 3.7; $t = -2.03$, $p < 0.05$). For the remaining outcome measures mean scores were better for patients treated with LSS, although the differences were not statistically significant.

### *iii) Patients' views of failed LSS*

There were no differences in terms of *age*, or *age at diagnosis* as a function of surgery group (see Table 1), but *time since diagnosis* was longest for patients with a secondary amputation ($Z = 8.40$, $p < 0.05$). The three groups did not differ in terms of level of education and type of employment. Patients with a secondary amputation (n = 8) were more likely to use a walking aid than those with a primary amputation or LSS ($\chi^2 = 7.19$, $p < 0.05$).

### *Interview data for patients with a secondary amputation*

*Decision making.* Interviews were conducted with 10 of the 11 patients undergoing secondary amputation. (One patient agreed to be sent a questionnaire but did not wish to take part in the interview). Five patients reported that they made the decision themselves to have their leg amputated. Three patients reported that the decision to amputate was made by the doctor. Two patients reported that the decision was made together. Examples of individual patients, illustrating the decision making process are shown in the appendix.

*Reason for amputation.* Decisions to amputate were invariably prompted by the occurrence of infections or complications which were no longer manageable. Six patients reported that they needed an amputation due to a persisting infection, whereas three patients explained that their body had rejected the metal. In all cases, these complications resulted in a substantial amount of pain, which contributed to the decision to amputate. One patient reported that the tumour had 'come back', and as a consequence the leg had to be amputated.

*Response to amputation after a failed LSS.* In general, patients were angry, afraid and disappointed about the need for amputation. Three agreed to surgery because they otherwise were afraid they would die. Most patients (8 of 10) did not regret that they had initially tried LSS, feeling that it had given them time to adjust to the amputation, or that they had become a stronger person generally.

Patients who reported that the doctor alone made the decision to amputate tended to be disappointed with the outcome; however, some of those who reported that they made the decision themselves were more positive (see appendix).

### *iv) Predicting QoL*

In order to examine the relationship between the dependent variable QoL and surgery, everyday competence and body image, regression analyses were conducted on the whole sample. Due to missing data on one or more of these variables our sample size was reduced by seven to 30 cases. Six were due to missing values for the variable QoL; however these missing values were random with respect to the other variables of interest in the analyses that follow.

Using Pearson's Correlation Coefficient we established that both everyday competence and body image were significantly positively associated with the dependent variable QoL (everyday competence $r = 0.74$, $p < 0.001$; body image $r = 0.75$, $p < 0.001$) and with each other ($r = 0.68$, $p < 0.001$).

Multiple regression analysis was subsequently used to test the hypothesis that everyday competence and body image are significant predictors of QoL independent of each other and of surgery. The first predictor entered was surgery, represented by a single dichotomous 'dummy' variable (where 0 = amputation and 1 = LSS). Everyday competence and body image were entered together in a second step.

Both body image (Beta = 0.4, $p < 0.05$), and everyday competence (Beta = 0.49, $p < 0.01$) were significant predictors of QoL, which together accounted for 67% of the variation in QoL scores (Table 3). Further investigation showed that while most of the variance of QoL explained by everyday competence and body image is shared, they each account for approximately 10% of 'unique' variance.

We next investigated whether the relationship between QoL and everyday competence varied as a function of surgery. We therefore added an interaction term to our regression model, (everyday competence multiplied by the dummy variable for surgery). This accounted for an extra 4.7% of the variance of QoL, ($R^2$ change = 0.047, $p < 0.05$). The introduction of the interaction effect into the model results in a reduction in the significance of the everyday competence main effect term (Beta = 0.37, $p < 0.05$) showing a stronger relationship between QoL and everyday competence for those

**Table 3.** *Results of regression analyses 1) The joint effects of everyday competence and body image on QoL, holding the variable surgery constant, 2) The moderating effect of surgery on the everyday competence-QoL relationship holding the effects of body image constant*

| | | Dependent variable—QoL | | | |
|---|---|---|---|---|---|
| Step | Predictors | b | $R^2$ | Adjusted $R^2$ | $\Delta R^2$ |
| 1 | Surgery[a] | –0.162 | 0.013 | –0.022 | 0.013 |
| 2 | Everyday competence | 0.489*** | | | |
| | Body image | 0.442* | 0.684 | 0.647 | 0.671** |
| 1 | Surgery[a] | –1.546* | 0.013 | –0.022 | 0.013 |
| 2 | Everyday competence | 0.375* | | | |
| | Body image | 0.405* | 0.684 | 0.647 | 0.671** |
| 3 | Everyday competence*Surgery | 1.455* | 0.731 | 0.688 | 0.047* |

n = 30, * $p < .05$, $p < .01$. Significance levels are for Fs at each step. β is standardized regression coefficient in final model.
[a] Dummy variable surgery: 1 = LSS, 0 = amputation with LSS compared with those treated by amputation as shown in figure 1.

### Discussion

Past attempts to determine differences in functioning between patients treated by amputation or limb salvage have yielded mixed results. However, our finding that patients treated with LSS report better everyday competence relative to patients with an amputation, is consistent with much previous work. Our findings extend those of Johansen *et al.*[8] who showed better physical functioning for LSS, but questioned the relationship with QoL.

Regardless of surgery, patients treated for a bone tumour report poorer QoL than expected from population norms. This was found for most subscales of the SF36, including physical function, physical role performance social function, vitality and pain. However, there were no significant differences in terms of mental health or emotional problems. Thus, despite compromised physical function, these patients do not report major emotional problems. These findings broadly replicate our previous research comparing outcomes of young people treated with successful LSS[10].

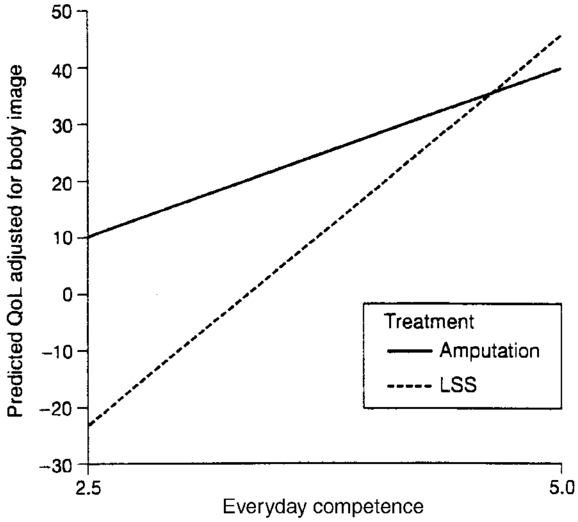

**Fig. 1.** *Relationship between predicted QoL (adjusted for Body Image) and Everyday competence, moderated by surgery (LSS vs amputation).*

From a clinical perspective, patients who experience failed LSS pose special problems. Negative experiences with LSS may mean that they are unfit prior to surgery, or resentful of pain experienced trying to adjust to LSS. As would be expected, circumstances leading up to the amputation differ, although most patients reported that the reason for the amputation involved persistent infection, the 'body rejecting the metal' or the 'tumour having come back'. According to patients, decisions were made by the surgeon alone, by themselves alone, or was a result of some joint negotiation. In practice, it is impossible to know now what actually happened. However, our data suggest that patients who accept responsibility for the decision tend to be more satisfied and better adjusted on interview, though the sample was inevitably small. These results also fit with other work that suggests that improved outcomes are associated with greater perceived personal control[16]. Of special interest is that 8 of 10 patients did not regret initial LSS but felt the time gained had allowed them to come to terms with secondary amputation.

The first regression analysis showed that QoL is predicted by body image and everyday competence, with better body image and everyday competence associated with greater QoL. It is important to note in the second regression the predictive value of the interaction term between everyday competence and surgery, which suggests that everyday competence is much more clearly associated with QoL for limb salvage patients compared with amputees. Thus, for patients undergoing LSS, QoL is very much bound up with everyday competence. It is possible that LSS patients have much higher expectations (or that others have higher expectations) of what they should be able to do, whereas amputees are more resigned to their limitations.

Our findings suggest that these patients are at risk of compromised QoL whether treated by LSS or amputation. Although most research in this area can be criticised on the grounds of small samples, together the results are pointing to greater difficulties for patients following amputation. Although amputation continues to be treatment of choice in high risks of local recurrence, there would seem to be strong arguments from the perspective of QoL to perform LSS wherever possible. Our data offer encouragement also to those concerned that secondary amputation may be specially upsetting for patients in that they face more extended treatment than if primary amputation was performed. The patients in our study were clear that the time gained prior to secondary amputation was beneficial in terms of helping them come to terms with secondary amputation. Our data also suggest that patients who felt more involved in the decision about secondary amputation were more accepting of the outcome. This points to the importance of patient participation in medical decision-making both for the immediate and longer term outcomes.

### Acknowledgement

This work was funded by the Cancer Research Campaign (CP1019/0101 and CP1019/0401). We would like to thank Carol Hughes for help recruiting patients to this study.

### References

1. Christ GH, Lane JM, Marcove R. Psychosocial adaptation of long-term survivors of bone sarcoma. *J Psychosoc Oncol* 1995; 13:1–21.
2. Greenberg DR, Goorin A, Gebhardt MC, Gupta L, Stier N, Harmon D, Mankin H. Quality of life in osteosarcoma survivors. *Oncol* 1994; 8:19–25.
3. Goorin AM, Abelson HT, Frei E. Osteosarcoma: Fifteen years later. *N Eng J Med* 1985; 313:1637–45.
4. Rosen G, Murphy ML, Huvos AG et al. Chemotherapy, en bloc resection and prosthetic replacement in the treatment of osteogenic sarcoma. *Cancer* 1976; 37:1–11.
5. Leplege A, Hunt S. The problem of quality of life in medicine. *JAMA* 1997; 278:47–50.
6. Eiser C, Grimer R. Quality of life survivors of a primary bone tumour: a systematic review. *Sarcoma* 1999; 4:183–190.
7. Rougraff BT, Simon MA, Kneisl JS, Greenberg DB, Mankin HJ. Limb salvage compared with amputation for osteosarcoma of the distal end of the femur. *Am J Bone Joint Surg* 1994; 76:649–656.
8. Johansen R, Nielsen OS, Keller J. Functional outcome in sarcomas treated with limb salvage surgery or amputation. *Sarcoma* 1998; 2:19–23.
9. Weddington WW, Segraves KB, Simon MA. Psychological outcome of extremity sarcoma survivors undergoing amputation or limb salvage. *J Clin Oncol* 1985; 3:1393–1399.
10. Felder-Puig R, Formann AK, Mildner A, Bretschneider W, Bucher B, Zoubek A, Puig S, Topf R. Quality of life and psychosocial adjustment of young patients after treatment of bone cancer. *Cancer* 1998; 83:69–75.
11. Eiser C, Cool P, Grimer RJ, Carter SR, Cotter IM, Ellis AJ, Kopel S. Quality of life following treatment for a malignant primary bone tumour around the knee. *Sarcoma* 1997; 1:39–45.
12. Ware JE, Snow KK, Kosinski M, Gandek B. *SF-36 health survey manual and interpreting guide*. Boston: Health Institute, New England Medical Centre.
13. Jenkinson C, Coulter A, Wright L. Short form SF 36 health survey questionnaire: normative data for adults of working age. *BMJ* 1993; 306:1437–1440.
14. Davis AM, Wright JG, Williams JI, Bombardier C, Griffin A, Bell RS. Development of a measure of physical function for patients with bone and soft tissue sarcoma. *Quality of Life Research* 1996; 5:508–16.
15. Davis AM, Bell RS, Badley EM, Yoshida K, Williams JI. Evaluating functional outcome in patients with lower extremity sarcoma. *Clin Orthop* 1999; 358:90–100.
16. Kopel SJ, Eiser C, Cool P, Grimer RJ, Carter SR. Assessment of body image in survivors of childhood cancer. *J Ped Psychol* 1998; 23:141–147.
17. Langer EJ, Rodin J. The effects of choice and personal responsibility for the aged: A field experiment in an institutional setting. *J Pers Soc Psychol* 1976; 34:191–198.

**Appendix.** *Quotes from patients on decision making process, and views about amputation after failed LSS*

| | Doctor's decision | Patient's views |
|---|---|---|
| 1. | They only took my leg off because the bone tumour had come back. | (Q: Were you disappointed at the time when you were having problems with the prosthesis?) In a way, yes. |
| 2. | He said, 'we might be able to cure it, but it could flare up again'. So he said, 'the best thing for you would be an amputation'. | |
| 3. | He said my body was rejecting it and that he'd have to amputate it. | I wish I still had my leg. |
| | Patient's decision | |
| 4. | Given the option of having another revision, which entailed the antibiotic being put in again and they were going to take skin from my shoulder and muscle from my shoulder, and I went no, you're not doing that and basically said to the doctor just to have it off. | But it would have saved a whole lot of trouble if I'd had it then. I probably would have been a lot more used to it than I think I am now. But I don't know. It was good in a way, because it built up my personality a wee bit. It just made me a stronger person. |
| 5. | I think they tried quite a few times to save it, that amputation is the last resort and I said, 'no, I think I've had enough, I want it amputated'. | (Q: Do you regret that decision?) No—because I know if I kept it I would have died. (Q: So you must have been quite disappointed with that?) I was, because I thought, well, they did actually tell me that 8 out of 10 are successful—2 aren't. But I think I made the right decision and I still do. |
| 6. | They said to me they could stabilise the infection, but not get rid of the infection. So I think I decided then to go for the amputation. | If I had gone straight for the amputation, I would probably have regretted going for the amputation straight away and not trying the internal prosthesis. |
| 7. | But eventually the infection was still there, because it was somewhere lurking about. So they had to take that one out, they took the same one out, and I asked the doctor to take my leg off. | |
| 8. | They took the infection away, but it just kept coming back. So they asked me if I wanted to stop in hospital and try and get rid of the infection or have my leg amputated. And, well, I didn't want to stop in hospital. So I had my leg amputated.—Well they asked me just before Christmas and I said, "Yes." | If I didn't have my leg amputated, I probably would have had a fear of dying because like my infection had just spread everywhere in my body to my lungs and that. I'd probably have the fear of me dying. But since I've had my leg amputated I don't feel that any more. I just feel normal. |
| | Joint decision between doctor and patient | |
| 9. | They decided to cut my leg off before it got any worse. The doctor in Birmingham made that decision, and I did as well at the end, because they told me there was nothing they could do. So I made the decision then, because it was very painful. I told them to get on with it, because I couldn't stand any more. | |
| 10. | Day by day I think the nursing staff and the doctors convinced me—I was on every antibiotic, they really did try hard. I honestly think they were waiting for me to make the decision, as opposed to them saying, 'right, we're taking your leg off tomorrow'. | |
